# Supplementary figures and images for: miR-625-3p and lncRNA GAS5 in Liquid Biopsies for Predicting the Outcome of Malignant Pleural Mesothelioma Patients Treated with Neo-Adjuvant Chemotherapy and Surgery
Source: Noncoding RNA. 2019 Jun 17;5(2):41. doi: 10.3390/ncrna5020041 (PMC6631473; doi:10.3390/ncrna5020041)

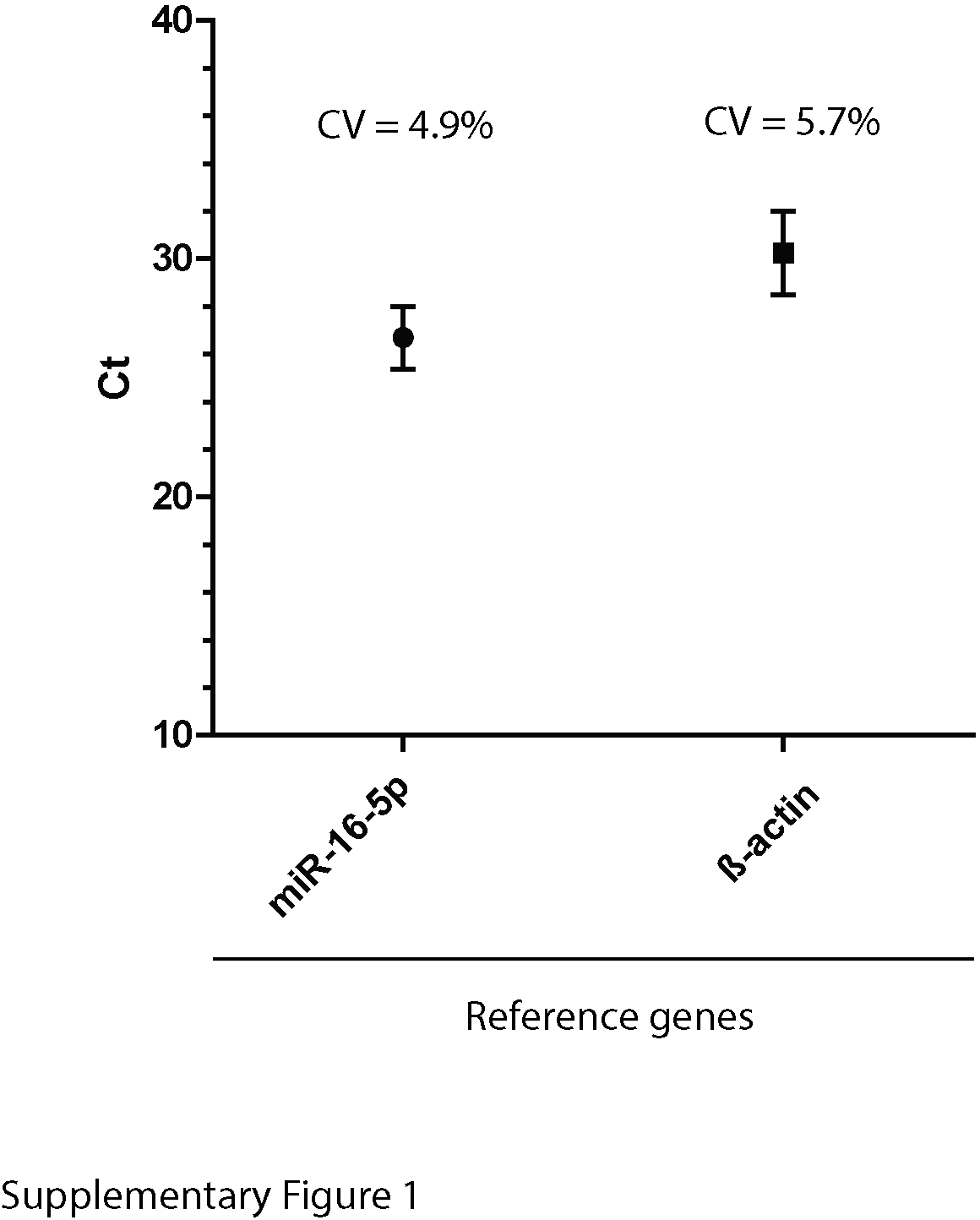

Supplement: Supplementary file 1 [file ncrna-05-00041-s001.zip › Kresoja et al Supplementary files/Kresoja et al Supplementary Fig 1 - reference gene.tif]

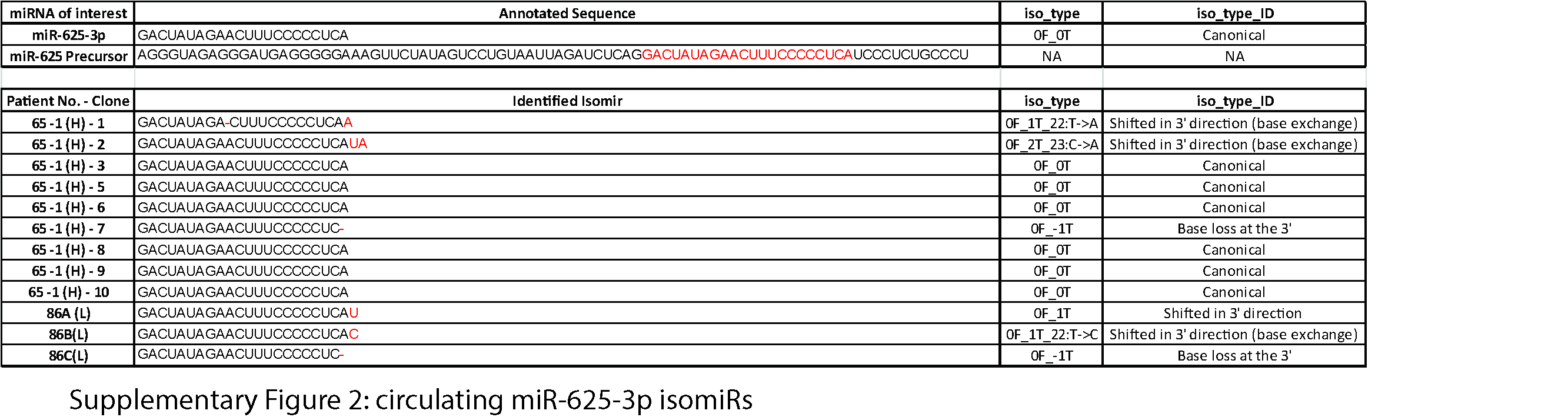

Supplement: Supplementary file 1 [file ncrna-05-00041-s001.zip › Kresoja et al Supplementary files/Kresoja et al Supplementary Fig 2 - isomiRs .tif]
